# Convalescent Camps

**Published:** 1918-10

**Authors:** Baily K. Ashford


### NOTES FROM THE FIELD SERVICE SCHOOL FOR MEDICAL OFFICERS OF THE AMERICAN EXPEDITIONARY FORCES

### CONVALESCENT CAMPS

Report of a lecture by Col. Baily K. Ashford, M. C., to the Field Service School for Medical Officers of the A. E. F.

*General Plan.* A convalescent camp should be carefully laid out and should be a barrack and not a tent affair. The site should be pleasant and healthful; above all it should be in the country away from all other centers, with plenty of room to expand. It should communicate by good roads with necessary centers, and should be laid out with a view to ornateness, with well turfed lawns, and abundant shade, with gardens and flowers, and with good walks which should include surroundings that would be pleasing to the eye and which would lead into the open country. These camps should accommodate from two thousand to three thousand men, who should be divided into companies of 250 each, under the immediate charge of experienced non-commissioned officers. There should be regular hours for meals, and such formations as are decided upon should be observed with military exactness. Great care should be taken at these places to provide for the men all military comforts and necessities with a minimum amount of the annoying petty details of a soldier's life. For example: the barrack itself. This should be attractively painted, with stained floor and stained rafters; with kalsomined walls and ceiling. It is an enormous advantage to have the floors waxed, or at least oiled. Beds are by no means a necessity; a straw mattress with a sufficient number of blankets, and a set of hooks and shelves above each man's head for his clothing and equipment being quite sufficient, *provided* the barrack itself is spotlessly clean, neat and attractive. The chill should be taken off the barrack by enough stoves, but the stuffy heat without ventilation, too often seen in such barracks, should be prevented. The men should sleep with the windows wide open. Both latrines and baths should be accessible and these should be as clean and as decent as the sleeping quarters.

There should be at each one of these convalescent camps an excellent quartermaster storehouse where an ample supply of clothing, field equipment, etc., should be kept at all times, so that no man need go dirty or slouchy, and every man when he goes away may be properly equipped to join his organization. There should be certain features about this quartermaster's storehouse that might with benefit be much more elaborate and detailed than in one of an ordinary depot type. Here considerable effort should be made to provide shoes that will fit the men. This work, begun by our medical corps in times of peace in our country and with such success, finds a peculiarly important mission to perform at these convalescent camps. In connection with these storehouses there should be a set of shops which could be used for the employment of men in the army who have become partially disabled for front line service — the so-called " B " men. Two such men should be permanently assigned here as tailors, and their shops should be used not only for the convenience of the men, but for the instruction of certain convalescents who will probably be unable to return to the fighting lines, and who may be advantageously placed in the lines of communication for the good of the army when they are sufficiently trained. This same policy should apply to all other industrial shops, which should form a feature of a convalescent camp. Thus a practical outcome of these camps will be a provision for training schools for artisans needed in the army. There should be, therefore, cobblers, tinsmiths, electricians, carpenters, blacksmiths, leather workers, laundrymen, cooks, musicians for bands, and others trained here.

The bathing, disinfecting and laundry systems should be unusually complete. In addition to the usual care taken to cleanse and disinfect the soldier upon his arrival, means should be provided to keep him clean and neat until he goes away. A delouser should be kept at this plant, which should be central and accessible; and the belongings of all men should be disinfected once a week. In addition to showers it is well to afford other varieties of baths. For instance, we noted in some of the convalescent camps that cement baths had been sunk in the floor for about a foot, underneath the shower, and that these baths were curtained off and made individual; both hot and cold water were available. It is well to have also a room for a Turkish bath; but the men should be made to go in groups to prevent accident, and a cool or temperate shower should be exacted of every man bathing thus before leaving the establishment. The baths should have attendants well versed in skin disease to pick out men who are lousy, who have the itch, or some other contagious disease of the skin. Fresh clothing should be freely supplied, as well as soap and towels. There should be a good barber shop in connection with this bath where men should not be required to pay for the cutting of their hair, although a small charge could be made for shaving and shampooing. A good chiropodist should also be on duty at the baths.

Above all, these baths should be scrupulously clean, and the men should be shown that the army can and will provide better bathing establishments than those for which high prices are paid in cities under conditions of peace. In this connection it is believed that the Section d'Hygiene Corporelle of the French could be used with advantage as the basis for American bathing establishment.

Some provision should be made for a good laundry, and this should be in charge of an experienced laundryman. No women should be employed at this place. It is believed that the sterilizing of clothing could best be accomplished either by the Herscher apparatus of the French or the Fodden Thresh of the English. By this time there may be some steam sterilizing apparatus on the plan of the Kny-Scheerer made in our own country.

*Feeding the Men.* The feeding of the men requires a very highly specialized department. The kitchen should be a large one, well ventilated, attractive, and furnished with a sufficient number of stoves to permit the cooks to devote themselves to specialties. There should be stoves for stews, stoves for baking, stoves for pastry, pies, cakes, etc., each being separate and each having its own cook. In this way there is great improvement in the quality of the food.

It was brought very forcibly to my attention that the method of the British at Boulogne in feeding the men should be copied in the interest of economy in our own service. All men should be given to understand at the outset that they can have all that they want to eat, and just as many helps as they want, but that it is their duty never to take more on their plates than they think will satisfy their appetites, and that they are sure they are going to eat. The kitchens adjoin the dining hall, a long counter for serving the men with food being interposed.

The men are introduced into the dining hall by companies, five minutes apart, instead of sitting them all at once. On entering the hall each man is given eating utensils, cups, plates, and paper napkins from the store of the mess hall. Armed with these he proceeds, after the manner of a Childs' restaurant, to the counter mentioned. By enlisting the co-operation of the men to the end that no more food will be taken on the plate than is needed, I was told by the commanding officer of one of the camps that ten thousand pounds a year was saved in food which had previously been wasted by the portioning out of fixed amounts to each man.

In connection with this kitchen there are two important side issues. One is the convalescent camp garden. In general terms a convalescent camp should produce all the vegetables it needs for its own support, but this garden should be utilized for the employment of men who will not go back to the firing line, rather than for the men who simply need to be rapidly built up for duty at the front. The distinction is thus made for the following reason: men who are to go back in a short time to the conditions of trench warfare should not be allowed to browse about too long in agricultural pursuits; their military life should be kept before them at all times. As one commanding officer of these camps states, " It seems like when they get into a garden they get to thinking what a terrible thing war is ". We don't mind this in the case of the permanent base men, but we don't want to make our men who have to go back to the front feel bad unnecessarily.

The other adjunct is the conservation of food.

First, *Fat Conservation*: This is important for two reasons: (1) it provides suet for cooking, and (2) it serves as a source of much material for certain munitions of war. The first necessity is subserved by the saving and rendering of fat trimmed from meat and taken from plates of men who have not consumed all that has been served them. The second is obtained from the fats of the kitchen waste, the garbage, and the scourings from kitchen sinks. This non-edible fat is rendered and is shipped in 300 pound casks to a salvage depot. The English render these fats on Soyer stoves which are excellent for the purpose. The important part of this conservation is that the organization is paid a few cents a pound by the Ordnance Department for all fat thus saved for munitions.

Second, *Conservation of Food Scraps*: An enormous amount of meat and bread can be conserved for the making of hash, rissoles and other dishes. It is noteworthy that these big kitchens have very few garbage cans— only one or two for a kitchen serving two to three thousand men.

Third, *Conservation of Solder from Tins*: At several convalescent depots we saw a large melting pot into which were thrown all tin cans. A hot fire was lighted two or three times a day and the solder allowed to run out through a funnel in the bottom. This was an important source of revenue for the camp, as the lead was sold in sticks of solder to the Government.

These camps require the most exacting sort of a sanitary officer. They should be models of sanitation. There should be night urinals placed between all barracks and, as before referred to, the latrines should be as perfect as it is possible to make them. Personally, I believe that the best possible disposition of feces at these camps is by incineration, and there is no objection at all to having these incinerators used for kitchen waste as well as feces; it will depend entirely upon the cleanliness of the latrine as to how near it can be placed to a kitchen. The pail system was so run in the British service that never in all of my many visits to their institutions have I smelt feces or seen filth in connection with such a system. Of course, a good sewer system is best, but it will be rarely possible to attain. Fly-proof boxes for deep pits, after the American plan, is a possibility in certain terrain and is preferred by many British to their bucket latrine. The great point is that it is not only sanitation that we are after, but we are trying to preserve the sanitary conscience of our soldiers by appealing to their sense of decency. Therefore, these places should be always free from odor, always spotlessly clean, always provided with a comfortable seat and good paper with which the men may cleanse themselves. The urine should be allowed to flow away into a soakage pit and an orderly should always be stationed on duty in charge of the latrine and the incinerator serving it.

As to the amount of fuel required for incinerator, as will be seen from many papers issued by this school, that quantity is negligible. With a good camp refuse conservancy system, two pounds of coke a day per incinerator for starting the fire is sufficient. But all camp refuse should be promptly dumped here daily.

The morning ablutions of the soldiers should be provided for by ablution benches, under cover, and with hot water for shaving. These can be near the latrine and the hot water obtained from pipes running through the fire box of the incinerator. In fact a wash house for hands and face is best located in this vicinity.

It should be emphasized that there is no part of the up-keep and the daily service of a convalescent camp which should not be run by the convalescents themselves; gradually a permanent detail will be formed from P. B. men who cannot go back to the front, men who have shown themselves to be particularly well prepared for identification with one or another of the services in the camp, and who will always serve as nuclei around which men for these services will be grouped. The commanding officer will inculcate in these men, in addition to the strictest regard for military discipline, a decent regard for the ornate, and, as a result thereof, for the clean and neat. It should be deliberately taught men that it is not enough merely to have the " rough stuff ", the bare necessities; that men should improve their condition; that they should keep themselves and their surroundings clean if they expect to keep well; that they should keep their initiative, their ambition, their imagination active; that to make their barracks, their reading rooms, their camp, attractive to the eye is to preserve those illusions of life which are necessary to make them contented and happy.

*Entertainments.* It should be noted that all too frequently the poorest form of entertainment for a soldier is the formula which somebody else has provided him to enjoy himself by. The most perfect entertainments that we have seen since we have been in France, generally vaudeville it is true, but some serious concerts, have been provided by the soldiers. The programs they have made up themselves from jokes which nobody ever wrote and from bits of waggery which you do not always find among professional comedians. The American people are especially gifted with a keen sense of humor, and we never see any humor keener than that displayed by our own healthy and care-free soldiers at the front. Civil societies who have taken unto themselves the diversion of the soldier can do their best work at these camps by providing the wherewithal for the soldier himself, and quite in his own way, to furnish his own amusement. Such entertainments "hand made" by the soldier himself, plus the excellent ones provided so lavishly by our patriotic professional artists, musicians, and orators, work marvels in keeping a normal and healthful psychology among our men.

For a camp of 2,500 there should be at least one drum and fife corps, two brass bands, one orchestra, and one mandolin, guitar and banjo club. By furnishing the instruments for these bands there never will be a dearth of musicians, and, as has been intimated previously, these camps can be a source of unborn bands for military services. The English made the great mistake of losing a great many of their bands, and in a general way it can be said that with music and healthful theatrical entertainment the spirits of the men never flag. Bands have an enormous influence in reviving the drooping spirits of men, and it is at these camps that such an influence is most needed. There should be some systematic effort, however, to train bands here, and just as there are trainers for physical exercises (to be spoken of later) there should be trainers in music and theatricals. There should be always plenty of music; the drills, games, etc., should be accompanied by music; there should be a drum and fife corps for parade, a band for retreat, and an orchestra for evening entertainments and dancing. There should be some *music*, good music, and not always merely a more or less agreeable noise. In addition to the amusement offered by bands, systematic effort should be made to draw out the men to take part in the theatrical entertainments above referred to. This requires a considerable amount of tact on the part of the commanding officer and really should receive the constant attention of some one person, because sometimes the most talented entertainers will go clear through a camp without displaying an iota of native ability, which they have to a very high degree, if properly brought out. Some of the best comedians we have seen have been quite accidentally discovered at these places. For these theatrical entertainments and minstrelsy, to which the American soldier takes very kindly, costumes should be freely supplied by the same patriotic civil societies who furnish the instruments for the bands.

Nor should these societies neglect to provide club rooms for non-commissioned officers and for men, reading rooms, writing rooms, libraries, and a good gymnasium where manly sports can be taught and practiced — boxing, wrestling, fencing, etc.

A sporting goods exchange should be provided where golf clubs, tennis rackets, bats, balls, and all the paraphanalia that indoor and outdoor sports require can be obtained. Some of the more expensive things should be rented ; some things, like tennis balls, base balls, etc., sold.

This is an enormously important feature of these camps, and here it is that these civil societies can be seen at their best. For instance, it is believed that in the exercises and the games which they are taught, regular physical trainers should be employed rather than army sergeants, for the simple reason that when men are being drilled by an army sergeant or any one to whom they are in a military sense beholden, the tension of military discipline at these games, after the manner of a drill, places the muscles in abnormal positions and under tension instead of relaxing them and freeing them from strain, which should be the object in exercising men for their physical good. These trainers should be good mixers and must learn how to tell when a man is bored or tired. Before the period of physical tire comes the mental tire should be recognized, and if it cannot be gotten rid of by personal address the man should be switched rapidly to some other form of game or exercise. These games and exercises should be held at fixed hours, and medical officers on duty at the camp should always be on hand among the groups to see that everything goes well. Regarding this participation of civil societies, it is believed that it is the manifest destiny of the Red Cross to take hold of this matter inasmuch as this is a medical unit and is being used to return the soldier fit to the lines ; in other words, this is a good opportunity for the Red Cross to cooperate actively with the medical corps, and not merely to furnish candy and cigarettes. For this purpose not more than two experienced and matter of fact middle-aged women of the Red Cross society should preside in a hut in which indoor games, checkers, whist, billiards, etc., could be conducted, little tables for writing provided, etc.

In other words, the whole spirit should be to make a man as comfortable as he can be and amuse him; at the same time he is being kept in a military atmosphere. There should not be too many orders, but general good discipline should be exacted, especially as regards cleanliness of person and surroundings, the rendering of proper salutes and the punctual attendance upon all formations. Loafers should always be kept busy at something.

*The Professional Care of Men* : Men should be given one general examination when they come in but they should not be examined too often — every five or six days is enough. Men who have disordered action of the heart should be gone over carefully, and if the surgeon is satisfied that there is nothing the matter with their hearts, of an organic nature, he should not repeat the examination again unless absolutely obliged to do so from symptoms. In other words, it is not wise to have attention drawn to organic diseases at these centers. In fact the whole atmosphere of treatment — medicine, disease etc., should be avoided. The spirit of the place should be " Here is the place for men to get fit in, and you need it. It depends on yourself how soon you fit yourself for the duties of a man ". As a matter of fact, in most of these convalescent camps there were very few actually in bed. In one (which cared for 3,000 men) there was only one hut which was known as a sick bay and that was empty but for one man. A powerful influence is the grading of men into the weaklings, those able to do something, and those who are getting husky and can do about everything. A spirit of self betterment is thus fostered by appeal to the pride of the man.

As a matter of fact these convalescent camps should be toward the rear, especially near large towns, although such centers will have to be placed near important bases for men who come to us sick from the States or who sicken after they get here. It seems that there are at least five kinds of institutions that could be grouped under this general heading " Convalescent Camps ", and that these institutions could all be administered along certain broad basic principles identical for all.

(1) A convalescent camp for the sick convalescents from disease, not venereal or neurological. All base hospitals should have or be near one of these.

(2) A convalescent camp to which men of (1) can be sent a few weeks after for hardening in order to enable them to return to the front. This camp could also receive men from the divisions direct who need a rest and change to prevent their breakdown.

(3) A camp for the correction of vicious postures and results of war wounds requiring re-education and prescribed exercises.

(4) A camp for the handling of war neuroses,

(5) A venereal labor camp.

